# FLCN Maintains the Leucine Level in Lysosome to Stimulate mTORC1

**DOI:** 10.1371/journal.pone.0157100

**Published:** 2016-06-09

**Authors:** Xiaochun Wu, Lingling Zhao, Zhi Chen, Xin Ji, Xianfeng Qiao, Yaping Jin, Wei Liu

**Affiliations:** Key Laboratory of Animal Biotechnology, the Ministry of Agriculture, College of Veterinary Medicine, Northwest Agriculture & Forest University, Yangling, Shaanxi, China; Yong Loo Lin School of Medicine, National University of Singapore, SINGAPORE

## Abstract

The intracellular amino acid pool within lysosome is a signal that stimulates the nutrient-sensing mTORC1 signalling pathway. The signal transduction cascade has garnered much attention, but little is known about the sequestration of the signalling molecules within the lysosome. Using human HEK293 cells as a model, we found that suppression of the BHD syndrome gene FLCN reduced the leucine level in lysosome, which correlated with decreased mTORC1 activity. Both consequences could be reversed by supplementation with high levels of leucine, but not other tested amino acids. Conversely, overexpressed FLCN could sequester lysosomal leucine and stimulate mTORC1 in an amino acid limitation environment. These results identify a novel function of FLCN: it controls mTORC1 by modulating the leucine signal in lysosome. Furthermore, we provided evidence that FLCN exerted this role by inhibiting the accumulation of the amino acid transporter PAT1 on the lysosome surface, thereby maintaining the signal level within the organelle.

## Introduction

The ancient mechanistic target of rapamycin (mTOR) signalling pathway is critical for cells to adjust their metabolism to the available nutrients. The central factor in this process is mTOR, a protein kinase whose targets include key regulators of protein synthesis. Not surprisingly, deregulated mTOR has been linked to multiple pathological conditions, including cancer and diabetes [1, 2]. mTOR can be incorporated into two protein complexes, mTORC1 and mTORC2. In a current model, the amino acid pool within lysosome serves as a signal to sequentially recruit Ragulator and the heterodimeric Rag GTPase complex with the help of lysosome-associated vacuolar-type H+ ATPase (v-ATPase). In its active form, the latter anchors mTORC1 on the lysosome surface, where it encounters Rheb and is activated [3–8]. Rheb itself is inactivated by the tuberous sclerosis complex (TSC), which is excluded from lysosomes in the presence of growth factors [9]. Thus, lysosome plays at least two roles in mTORC1 signalling. First, it provides a physical space where activation takes place; second, its content of amino acids activates the signalling. To date, most studies have focused on the cascade that transfers the luminal signal to mTORC1, but the sequestration of upstream cues within the lysosomal lumen remains poorly understood.

The genetic Birt–Hogg–Dubé (BHD) syndrome is characterized by benign skin tumours, lung cysts and a high risk for developing kidney cancers. The molecular basis of BHD was first associated with mutations of the folliculin (FLCN) gene at the beginning of this century [10–13]. The available evidence suggests that FLCN, often together with FNIPs (FLCN-interacting proteins), is associated with several cellular signalling pathways, including mTOR, AMPK, JAK/STAT [14] and TGF-beta [15, 16], where it participates in versatile cellular processes, such as cell adhesion [17, 18], membrane traffic [19], autophagy [20], the biogenesis of lysosome [21] and mitochondria [22], and other processes [23–27]. However, its molecular functions are not fully understood.

The relationship between FLCN and mTOR is unclear yet because mTOR can be either up- or down-regulated under loss-of-FLCN conditions in various model systems, suggesting that FLCN controls mTOR in a context-dependent manner [18, 21]. Using *Drosophila* as an animal model, we previously found that the fly FLCN (*DBHD*) mutants grew slowly and died as small larvae, a consequence that is similar to that of protein starvation or impaired mTOR signalling. Surprisingly, supplementation with high level of leucine, but not other amino acids, in the medium markedly rescued the growth phenotypes. Furthermore, the addition of rapamycin, a specific inhibitor of mTOR, to the same leucine-rich medium failed to rescue the phenotype [28]. These results suggest that loss of *DBHD* sensitizes flies to the leucine signal that stimulates mTOR. However, the underlying mechanisms remain to be elucidated. More recently, two groups independently reported that in a mammalian cell system, FLCN bound and activated the Rag complex on the lysosome, which was necessary to anchor mTORC1 [21, 29]. These works demonstrate that FLCN is involved in the signal transduction process, and lysosome is the site for FLCN to perform this function.

In this paper, we report a novel, more upstream role of FLCN in the leucine-stimulated mTORC1 signalling. Specifically, FLCN controls the leucine signal in lysosome. Using human embryonic kidney 293 (HEK293) cells as a model, we found that lysosomal leucine was reduced upon FLCN suppression, causing decreased mTORC1 activity. Supplementation with high leucine reversed both consequences. Conversely, elevated FLCN levels enable cells to sequester leucine within lysosome and activate mTORC1 under amino acids starvation conditions. We also provide evidence that FLCN exerts this function by preventing the accumulation of the transmembrane amino acid permease PAT1 (Proton-Assisted Amino Acid Transporter 1, or slc36a1) on the lysosomal surface. Our work demonstrates that the FLCN-mediated modulation of the leucine level in the lysosome is an important mechanism to regulate mTORC1 signalling.

## Materials and Methods

### Antibodies and chemicals

Antibodies: pS6K1 (T389, #9205), S6K1 (#9202), FLCN (rabbit monoclonal, #3697) and histone H4 (#2935) were obtained from Cell Signalling Technology (Danvers, MA); FLCN (mouse monoclonal, #271558) and bafilomycine A1 was from Santa Cruz Biotechnology; LAMP1 (H4A3) and GFP (12A6 and 8H11) were purchased from Developmental Studies Hybridoma Bank; calnexin (#22595) and HA (#9110) was obtained from Abcam; GFP (A11122) and all fluorescent secondary antibodies were purchased from Life Technologies; amino acids were from Sigma Aldrich.

### Plasmids

Human FLCN cDNA was kindly provided by Schmidt, L.S. Human PAT1 and LAMP1 cDNA were both obtained from a cDNA pool generated by the reverse transcription of total RNA extracted from HEK293 cells. For ectopic expression, FLCN with an HA tag on the 3’-end was amplified by PCR and cloned into the pCDNA3.1 vector (EcoRI/XhoI); PAT1 (or LAMP1) cDNA was cloned into pEGFP-C1 (or pEGFP-N1, in XhoI/EcoRI), which encodes an EGFP-PAT1 (or LAMP1-EGFP) fusion protein. All transgenes were expressed under the ubiquitous CMV promoter. The resulting constructs were confirmed by sequencing.

### Cell culture and transfection

Cells were cultured in DMEM (Life Tech.) supplemented with 8% fetal bovine serum (FBS), penicillin and streptomycin. FLCN-HA, EGFP-PAT1 or LAMP1-EGFP plasmid was introduced into HEK293 cells using the TurboFect transfection reagent (Life Tech.). Stable cell lines were selected on the bases of the neomycin resistance. For RNAi experiments, RNAiMax diluted in OptiMEM (Life Tech.) was used to deliver siRNA according to the manufacturer's instructions; shRNA sequences were cloned into the expressing plasmid (pCD513B-U6) and co-transfected with the helper plasmids (GAG, REV and VSV-G) into HEK293T cells using TurboFect transfection reagent (Life Tech.). Purified and concentrated virus was used to infect cells with puromycin selection. To confirm the data of the knockdown experiments, we tested at least two RNAi of each target gene following published works and obtained similar results, including two siRNAs of FLCN (siFLCN-1 and 2 in ref. 21), two shRNAs of FLCN (shFLCN-1 and 2 in ref. 29), two siRNAs of PAT1 (si158 and si160 in ref. 30) and two siRNAs of PAT4 (si435 and si437 in ref. 30). After transfection, cells were allowed to recover for at least 36 hrs before the following treatment.

### Amino acid starvation/stimulation

The cells were rinsed once with phosphate-buffered saline (PBS) and incubated with either amino acid-free RPMI-1640 medium (US Biological, R8999-04A) supplemented with 8% FBS or DMEM medium supplemented with 8% FBS plus different amounts of amino acids for the indicated period of time. The following amino acid concentrations were used in this work (1X, 50X, in mM): Leu (0.8, 40); Arg (0.48, 24); Gln (3.99, 199); Lys (1.0, 50).

### Western blotting and coimmunoprecipitation

Cell extracts were subjected to protein separation using sodium dodecyl sulphate-polyacrylamide gel electrophoresis (SDS-PAGE) and then electrophoretically transferred to PVDF membranes (Millipore). The membrane was blocked with Tris-buffered saline containing 0.5% Tween 20 (TBST) and 5% nonfat dry milk powder for 1 h. The incubation with primary antibody was carried out at 4°C overnight, and the secondary antibody incubation was performed at room temperature for 1 h. After extensive washes with TBST, the blot was visualized by enhanced chemiluminescence (Advansta, USA). The quantification analysis was performed using the Bio-Rad Quantity One software.

For the coimmunoprecipitation experiments, HEK293 cells were rinsed once with ice-cold PBS and lysed with NP40 lysis buffer (0.5% NP-40, 25 mM Tris, 200 mM NaCl, 200 mM KCl, 1.5 mM MgCl2, 0.5 mM PMSF, 1 mM EDTA, 5% glycerol, and protease inhibitor cocktail, Roche, pH7.4), followed by centrifugation at 10,000*g* for 10 mins at 4°C. The supernatant were immunoprecipitated with either anti-GFP polyclonal antibody (Life Tech.) or anti-FLCN. Protein A/G PLUS-Agarose (Santa Cruz) was then added to the above cell lysate with rotation at 4°C for 2 hrs. The immunoprecipitated proteins were analysed by western blotting.

### Immunofluorescent staining

The cells were grown on glass coverslips. After fixation with 4% formaldehyde in PBS for 10 mins. The cells were rinsed once with PBS and permeabilized by incubation with PBST for 5 mins (0.1% Triton X-100 in PBS). Incubation with either primary or second antibody was performed at room temperature for 2 hours. Nuclei were counterstained with DAPI (1 μg/ml in PBS, Sigma). Images were captured using confocal microscope (Nikon A1R-si). To quantify the colocalizations of FLCN and LAMP1, two-channel stacks of unedited images were analyzed using the Nikon NIS-Element software and the Pearson's correlation coefficient was calculated. For each sample, at least 10 cells in each of three repeated experiments were analyzed. The t-test was used to assess the results, ±SEM.

### Fly feeding experiment

Fly stocks: esg-Gal4, da-Gal4 and lsp-Gal4 were obtained from the Bloomington stock centre; UAS-DBHD was described previously [28]. Flies were routinely raised on normal medium (8% sugar, 10% corn flour, 1.5% baker’s yeast, 1% agar, 0.4% Propionic Acid and 0.1% Nipagin) under standard conditions (25°C, ~60% humidity). Each Gal4 line was individually crossed with UAS-DBHD. Once females started to lay eggs, they were transferred to fresh starvation food (0.3% yeast) or starvation food containing 1 μM rapamycin and allowed to lay eggs for 24 hours. The newborn flies were counted each day. Heterozygote flies eclosed from the same cross (no overexpression) were taken as the control. Typically, a wild-type embryo fed normal food requires approximately 7 days reaching adulthood.

### Lysosome purification and amino acid quantification

Crude lysosomes were isolated using a Lysosome Isolation Kit (LYSISO1, Sigma-Aldrich, USA). For each sample, five dishes (90 mm) of cells grown to 90% confluence were collected by centrifugation at 600*g* for 5 min (~1.5 X 108 cells in total). The following steps were carried out on ice or at 4°C. The cells were resuspended in 200 μl extraction buffer and homogenized with 5 gentle strokes in a 2 ml Dounce glass tissue grinder. Normally, this treatment lyses approximately 80% of cells (checked under microscope). After centrifugation at 1000*g* for 10 min, the supernatant was collected, and the pellet was homogenized for another four rounds. The final pellet was considered the “nuclei” fraction, which contained mostly nuclei and un-homogenized cells. Supernatants from all five homogenizations were then collected (~1 ml) and centrifuged at 20,000*g* for 20 min. The resulting supernatant was referred to as the “cytosol” fraction. The pellet was resuspended in 200 μl of extraction buffer to yield the crude lysosome fraction (CLF), which was checked by immunoblotting of LAMP1 and LysoTracker Red staining.

The lysosome was further purified from the CLF through density gradient ultracentrifugation method following a previous report [31]. The density gradient was prepared by sequentially adding different concentration of Optiprep Density Gradient Medium Solution (Sigma) diluted with 2.3 M of sucrose (bottom to top: 27, 22.5, 19, 16, 12 and 8%) into a centrifuge tube. The CLF was firstly mixed with 300 μl of 19% Optiprep Density Gradient Medium Solution and carefully placed on top of the density gradient. Centrifugation was carried out at 150,000*g* for 4 hours at 4°C. According to the previous report [31], we collected the upper 50% (v) resulting solution, mixed it with an equal volume of PBS and centrifuged at 180, 000*g* for 30 min. The pellet (~20–25 μl) was washed once with PBS and resuspended in 20 μl of ice-cold extraction buffer containing proteinase inhibitor and 8 mM CaCl2 to destroy the residual mitochondria contaminant. After incubation on ice for 15 min, it was centrifuged at 5000*g* for 15 min. The supernatant was collected and centrifuged at 180, 000*g* for 30 min 4°C. The pellet was the purified lysosome and lysed with 50 μl of lysis buffer containing phosphatase inhibitor, proteinase inhibitor and PMSF. Cellular fractions were examined by immunoblotting for LAMP1. Calnexin (ER) and histone 4 (nucleus) were taken as two indicators of the purity of lysosomal fractionanation. The amount of lysosome (or cytosolic fraction) was normalized to the total protein content of the purified lysosomes (or cytosol). The free amino acid concentrations were measured using liquid chromatography-tandem mass spectrometry (LC-MS/MS) at the Center for DNA typing of the Fourth Military Medical University, China.

## Results

### High leucine supplementation reverses the down-regulated mTORC1 in FLCN-deficient HEK293 cells

**Fig 1:**
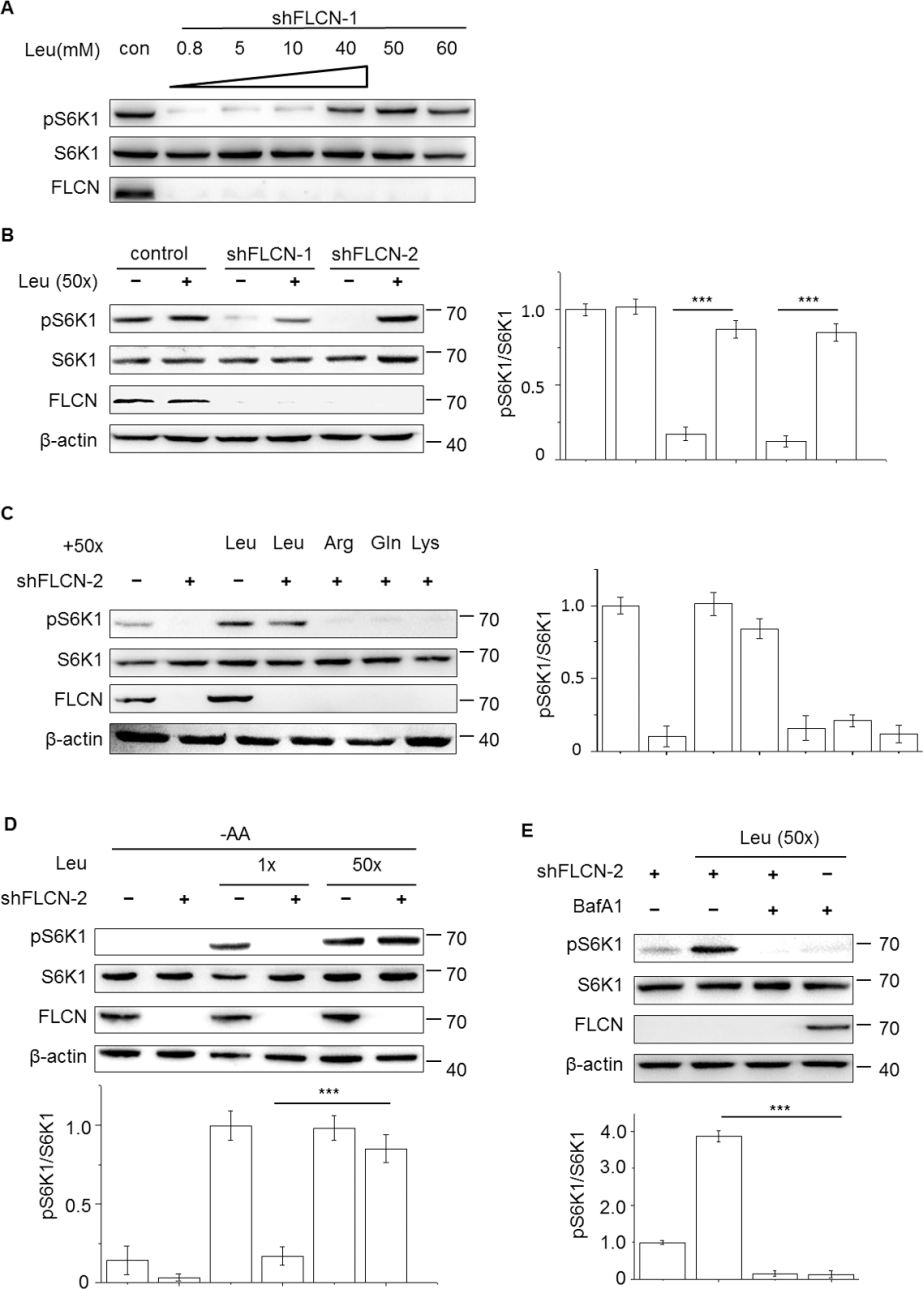
High leucine antagonizes FLCN-deficiency to stimulate mTORC1. (A-C) Cells were allowed to express the FLCN shRNA (shFLCN) for 48 hours, followed by stimulation with different doses of the indicated amino acids for another 2 hrs. In A, the treatment with 0.8 mM of leucine means cultured with complete culture medium. (D) Cells were starved with amino acid-free RPMI 1640 medium for 1 hour, stimulated with the indicative amount of leucine for additional 2 hrs. (E) The stimulation with leucine, BafA1 (0.1 μM) or in combination was performed for 2 hrs. In the quantitative analyses, the gray values of pS6K1 were normalized to that of S6K1. Error bars denote the mean ± S.E.M. (n = 3 repeated experiments); *** P<0.001, Student’s t-test. In all the experiments, representative pictures from at least three repeated experiments were presented.

In our previous *Drosophila* work, we demonstrated that a high level of dietary leucine, but not other tested amino acids (including tryptophan, arginine and glutamine), considerably rescued the slow growth phenotype of *DBHD* mutants [28]. However, it is not known whether this mechanism is conserved in mammals. Recently, two groups reported that suppression of FLCN inhibited mTORC1 in both insect and mammalian cells [21, 29]. We replicated these experiments and confirmed their discovery: in human HEK 293 cells, the knockdown of FLCN by expressing a virus-mediated short-hairpin RNAs targeting FLCN (shFLCN-1, ref. 29) suppressed the phosphorylation of S6K1 on threonine 389 (pS6K1), a marker of mTORC1 activity (**Fig 1A; lane 2**). On the basis of this experiment, we increased the amount of leucine in the culture medium as a supplement, attempting to reverse the down-regulation of mTORC1.

According to the manufacturer, the culture medium that we were using contained 0.8 mM of leucine (DMEM, Life Tech.). When this concentration was gradually increased up to 40 mM (50X) and combined with an extended incubation time (up to 2 hrs), the signal of pS6K1 was markedly maintained in the FLCN-depleted cells (**Fig 1A**). We confirmed this finding by using two different sets of shFLCN following high leucine stimulation treatment (**Fig 1B**).

During these experiments, we did not find notable phenotypes of the cells following high leucine stimulations (less than 2 hours of stimulation before harvesting the cells). Therefore, we used 40 mM (50X) of leucine in the following related experiments.

Leucine is potent stimulator of mTORC1. Excess leucine may induce strong mTORC1 activity to compensate for the reduction caused by FLCN-loss or mTORC1 can be triggered by a high leucine signal via an FLCN-independent pathway. However, both cases are somewhat unlikely because the pS6K1 signal in wild-type cells did not apparently increase, even in response to high leucine supplies (**Fig 1B**; compare lane 1, 2). Considering the similar rescues in the fly mutants that the FLCN gene was completely missing [28], it is probably a conserved mechanism that leucine can substitute for at least some FLCN functions to activate mTORC1. We also tested high levels of three other amino acids (50X), arginine, glutamine and lysine, and found that the rescue effect was specific to leucine (**Fig 1C**). This result was not completely unexpected because the FLCN-containing Rag complex has been shown to transfer signals from leucine, but not glutamine, to mTORC1 [32].

We also performed another dosage experiment by starving cells with amino acid-free medium and then added either basal or high doses of leucine. In wild-type cells, leucine at a basal dose (0.8 mM) has almost maximized the activation (**Fig 1D**, lane 3, 5). However, in FLCN-deficient cells, a basal dose resulted in much weaker signalling than a high dose (**Fig 1D**; lane 4, 6). Thus, the suppression of FLCN sensitizes cells to the available leucine for mTORC1 induction.

Upstream of the Rag complex, the lysosome-anchored v-ATPase maintains the acidic environment in the lysosome and is required to sense the amino acid signal to mTORC1 [6]. We found that bafilomycine A1 (BafA1), a specific inhibitor of v-ATPase [33], blocked mTORC1, even in the presence of a high leucine supply (**Fig 1E**). This finding implies that the stimulation of mTORC1 by high levels of leucine requires functional v-ATPase and probably also intact lysosomes.

### FLCN regulates the level of leucine in lysosome

The amino acid pool within the lysosome has been shown to be a direct signal to activate mTORC1 [6, 34]. Combined with the feeding experiment data in *Drosophila*, we proposed before that FLCN might modulate intracellular leucine, especially those in the lysosome, to control mTORC1 [28].

**Fig 2:**
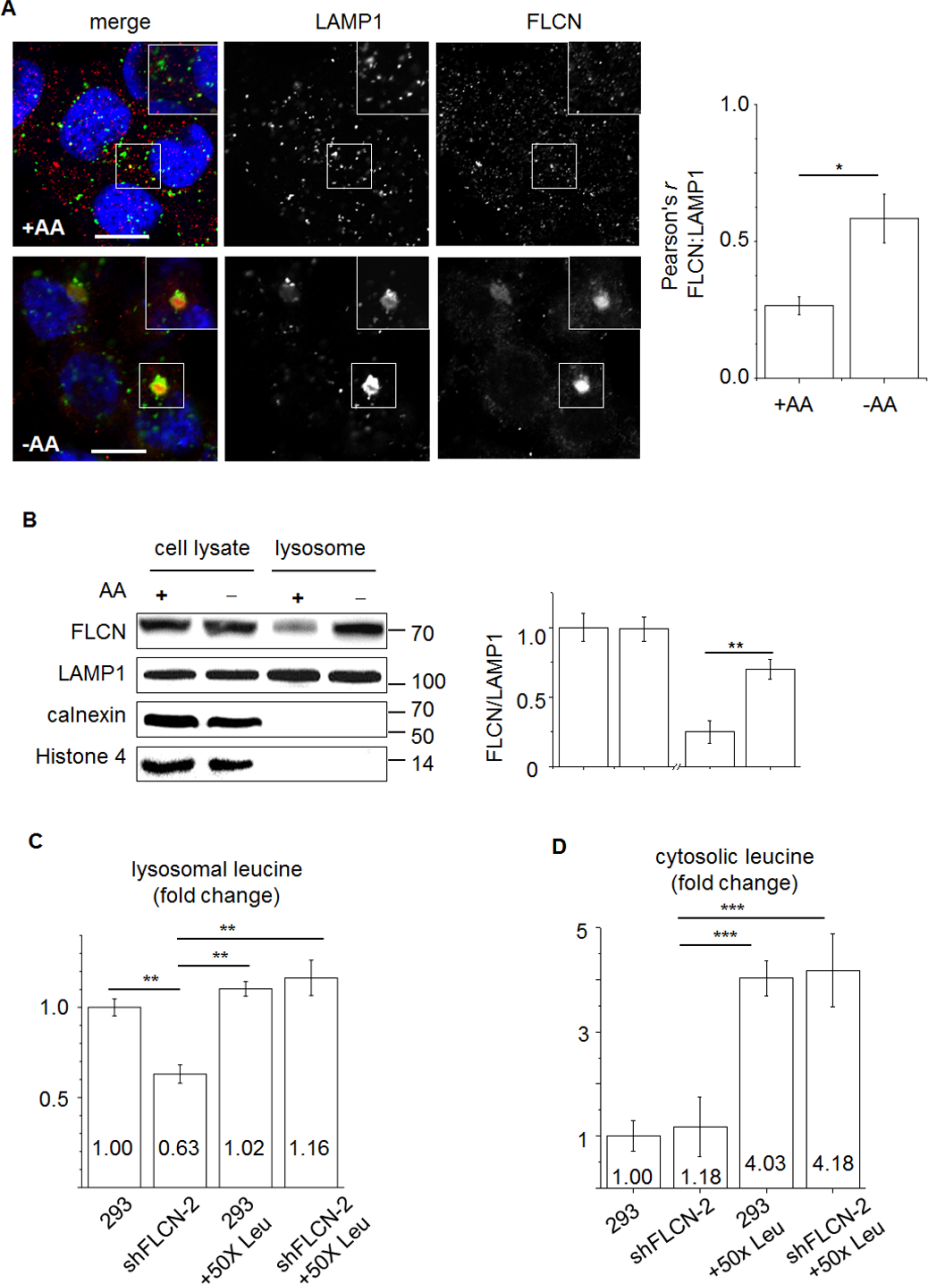
FLCN regulates the leucine level in lysosome. (A) Control (+AA) and staved cells (-AA, for 1 hour) were co-stained for FLCN (red) and LAMP1 (green). DNA was stained with DAPI (blue). Scale bar: 10 μM. Co-localization of FLCN and LAMP1 was measured using the Nikon NIS-Element software and shown by Pearson's correlation coefficient (Pearson's *r*). (B) Whole cell lysate or the purified lysosomes were analysed by western blotting. Each loaded sample of the purified lysosome was about 30% to that of the whole cell lysate (in total protein). Representative pictures from at least three repeated experiments were presented. (C, D) Quantification analysis of the lysosomal (C) and cytosolic (D) leucine. Data were obtained from three independent experiments and are shown as mean ± S.E.M. Numbers on the column are the relative values that have been normalized to that in control (1.0). * P<0.05, ** P<0.01, *** P<0.001, Student’s t-test.

This hypothesis implies that FLCN resides on the lysosome. Previously, two groups have provided immunofuorescent staining data showing that FLCN is localized on lysosome in a nutrient-dependent manner: FLCN was accumulated on lysosome upon starvation and was dispersed into the cytoplasm by restimulation with amino acids [21, 29]. We repeated this experiment and obtained similar observations: FLCN seemed to be evenly distributed within the cytoplasm in complete medium and became accumulated on the lysosome when amino acids were depleted (**Fig 2A**). Based on the immunostaining data, it is not clear if FLCN is located on the lysosome under the normal culture conditions. Therefore, we applied another approach by purifying the lysosome from cell lysate (see Materials and methods) and measured FLCN through western blotting. Consistent with the immunostaining data, there was a strong signal of the lysosome-anchored FLCN when cells were starved with amino acids, (**Fig 2B**; lane 4). Notably, we could also readily detect a relatively weaker signal even when cells were cultured in complete medium (**Fig 2B**; lane 3). This result supports that a small amount of FLCN on the lysosome is probably sufficient to activate mTORC1 (via Rags) in the presence of amino acid [21, 29].

After confirming its presence on the lysosome, we explored the influence of FLCN on the upstream cue, namely the amount of amino acids within the lysosome. To this end, we purified lysosomes and measured the luminal concentrations of free leucine. In three independent experiments, leucine was consistently reduced in the lysosomes from cells expressing shFLCN, which was efficiently blocked by supplementation with high levels of leucine in the medium (**Fig 2C**). These results suggest that FLCN maintains the leucine level in lysosome, which can be alternatively achieved by increasing the leucine supply in the environment.

The transport of leucine across cellular membranes was thought to be mediated through facilitated diffusion, meaning it moves down the concentration gradient with the aid of special transmembrane carriers. Under certain conditions, such as when the extracellular supply is high, the transport can also occur via low-affinity systems or passive diffusion [35–37]. Consistent with these alternative mechanisms, incubation with high amounts of leucine significantly increased its level in the cytosol of both wild-type and FLCN-deficient cells (**Fig 2D**). In contrast, the level of leucine in the lysosomes of the same population of wild-type cells only slightly increased. Accordingly, the level of pS6K1 was not markedly increased (**Fig 1A**). We conclude that the suppression of FLCN leads to decreased leucine in the lysosome, which is insufficient to fully activate mTORC1. However, high leucine supplementation results in its accumulation in both the cytosol and lysosome due to saturation mechanisms. As a result, the leucine signal in the lysosome strengthens sufficiently to activate mTORC1.

### Elevated FLCN renders cells resistant to starvation to activate mTORC1

We attempted to test this FLCN function from a different perspective by investigating the ability of ectopic FLCN to sequester more leucine within the lysosome. To this end, we generated several stable cell lines expressing FLCN-HA controlled by the ubiquitous CMV promoter, which were considered to be FLCN-overexpression systems in the following experiments.

**Fig 3:**
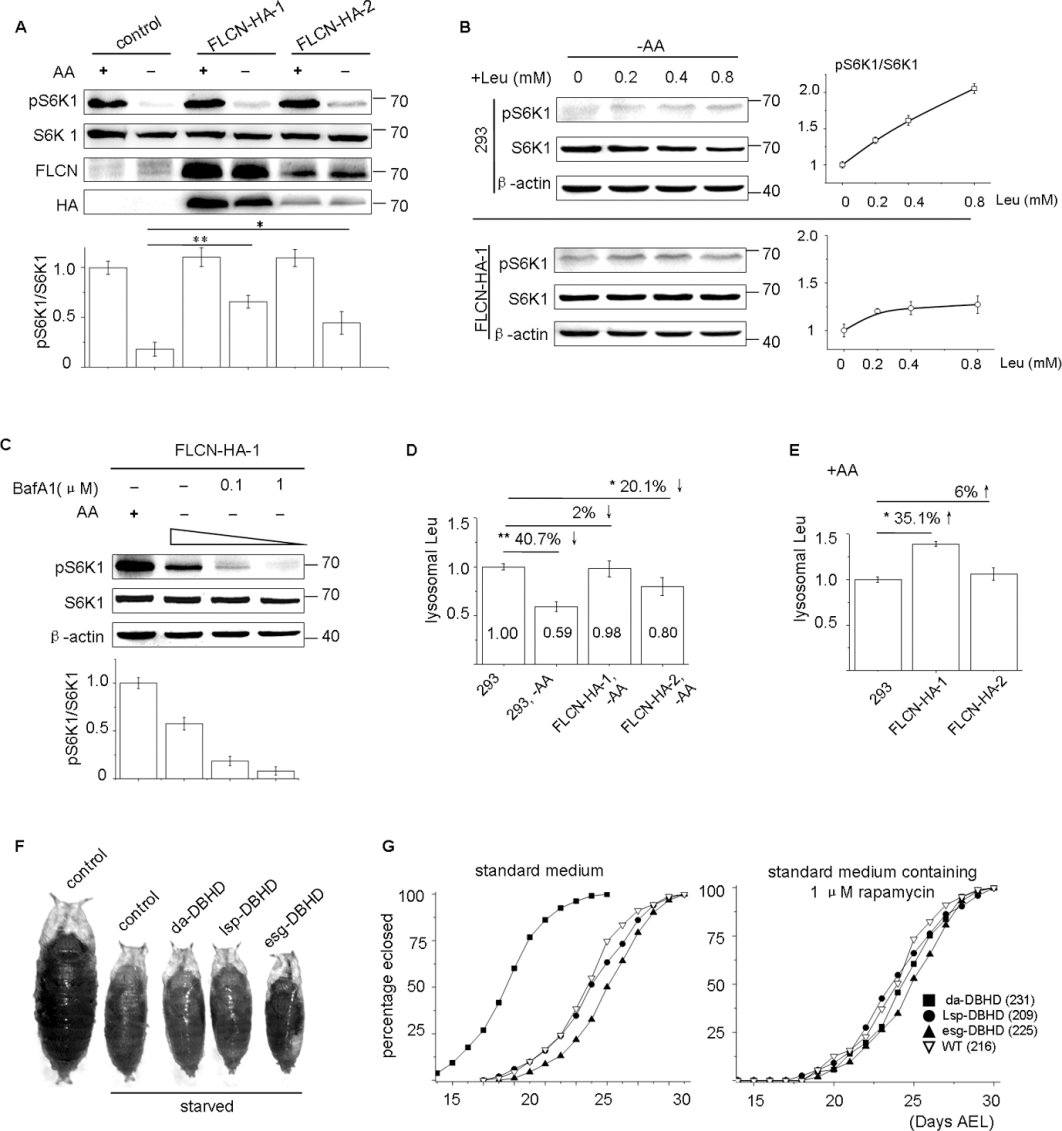
Ectopic FLCN renders cells resistant to starvation for mTORC1 induction. (A) Control (293) and two FLCN-HA stable cell lines were analysed by western blotting. Starvation was lasted for 1 hour. (B) Cells were starved for 50 mins, stimulated with different amounts of additive leucine for another 30 mins. (C) BafA1 inhibits the ectopic FLCN-stimulated mTORC1. (D, E) Quantitative assay of the lysosomal leucine. Note starvation reduces the lysosomal leucine, but to a lesser extent in the FLCN-HA cells (D); (E) in complete medium (+AA). (G) A picture showing the body size of fly pupae. Heterozygotes from the same crosses without ectopic expression were taken as the control. (H) Fly growth profiles. Error bars denote the mean ± S.E.M. (n = 3 repeated experiments); * P<0.05, ** P<0.01, Student’s t-test.

In complete culture medium, the pS6K1 signal was not markedly increased in cells expressing ectopic FLCN-HA (**Fig 3A**; lane 1, 3, 5). However, in amino acid-free medium, pS6K1 was decreased in both wild-type and two stable FLCN-HA cell lines, but the decrease was less pronounced in FLCN-HA cells (**Fig 3A**; lane 2, 4, 6). Thus, elevated FLCN enables cells to resist the shortage of amino acids for mTORC1 induction. In a dosage experiment, we re-stimulated the starved cells with different doses of leucine. In wild-type cells, the signal of pS6K1 parallels the concentration of supplemented leucine when it is below basal doses (<0.8 mM), implying that mTORC1 is sensitive to the leucine level under this culture condition. However, this sensitivity was clearly decreased in the FLCN-HA stable cell lines (**Fig 3B**). In addition, BafA1 inhibited mTORC1 in the FLCN-HA cells in a dosage-dependent manner, suggesting v-ATPase acts downstream of or in parallel with the overexpressed FLCN (**Fig 3C**).

To examine the correlation of the above data with the levels of lysosomal leucine, we measured the abundance of leucine in purified lysosomes. Taking the value in wild-type cells cultured in complete medium as a reference, we found about 41% reduction in lysosomal leucine after amino acid deprivation. In contrast, this number decreased to 2% and 20% in the two stable FLCN-HA lines (**Fig 3D**). Even cultured with complete medium, the FLCN-HA cells contained more lysosomal leucine than wild-type cells (**Fig 3E**). However, this increase seemed to be more prominent in amino acid-free medium (**Fig 3D**), probably because there are more FLCN proteins on the lysosome under starvation.

These data suggest that ectopic FLCN promotes the accumulation of leucine within the lysosome, which decreases the sensitivity of these cells to leucine shortages in the environment and facilitates mTORC1 induction. Previously, Baba et al. observed that a patient-derived, FLCN-mutated cell line stably expressing ectopic FLCN was resistant to amino acid deprivation to activate mTORC1 (Fig 7B in ref. 24). Because they also expressed FLCN using a constitutive promoter, we suspect that their cell line might act similarly with our FLCN-overexpression systems.

### Overexpression of DBHD promotes the growth of Drosophila in starved environment

To test the discovery in multicellular organisms, we used *Drosophila* as an animal model and overexpressed the fly FLCN gene (*DBHD*) using three different tissue-specific Gal4 driver lines, including da-Gal4 (ubiquitous), lsp-Gal4 (fat body, the nutrient storage organ) and esg-Gal4 (intestinal progenitor cells). When fed with standard medium, most embryos developed into adults within one week without apparent abnormalities, indicating that excessive *DBHD* does not affect general morphogenesis and growth. When fed a low-protein food (20% of the level in the normal recipe), all newly eclosed flies shank in size (**Fig 3F**) and required an extended period of time to reach adulthood (nearly doubled). Both were typical responses to starvation or impaired mTOR signaling [38]. Interestingly, flies expressing a high level of FLCN ubiquitously (da-DBHD) showed a growth advantage over other flies, as exhibited by a shorter developmental time (**Fig 3G**). Nevertheless, this effect was suppressed by administration of rapamycin (**Fig 3G**), suggesting that the growth advantage is caused by relatively high mTOR activity.

### FLCN and PAT1 antagonize each other to control mTORC1

Lysosomal leucine levels can be reduced by either impaired protein breakdown or accelerated loss. As FLCN is located on the lysosome, it may regulate the efflux of leucine, a process that normally requires it to be loaded onto membrane-anchored transporters. We searched the literatures and considered PAT1 as a good candidate of this transporter. PAT1 was initially identified as a lysosome-associated membrane protein whose function was to export amino acids into the cytosol [39]. In fact, previous work has shown that the overexpression of PAT1 promoted the release of amino acids from lysosome and inhibited mTORC1 [6]. Based on these results, we investigated the involvement of PAT1 in the FLCN-mediated mTORC1 signalling.

**Fig 4:**
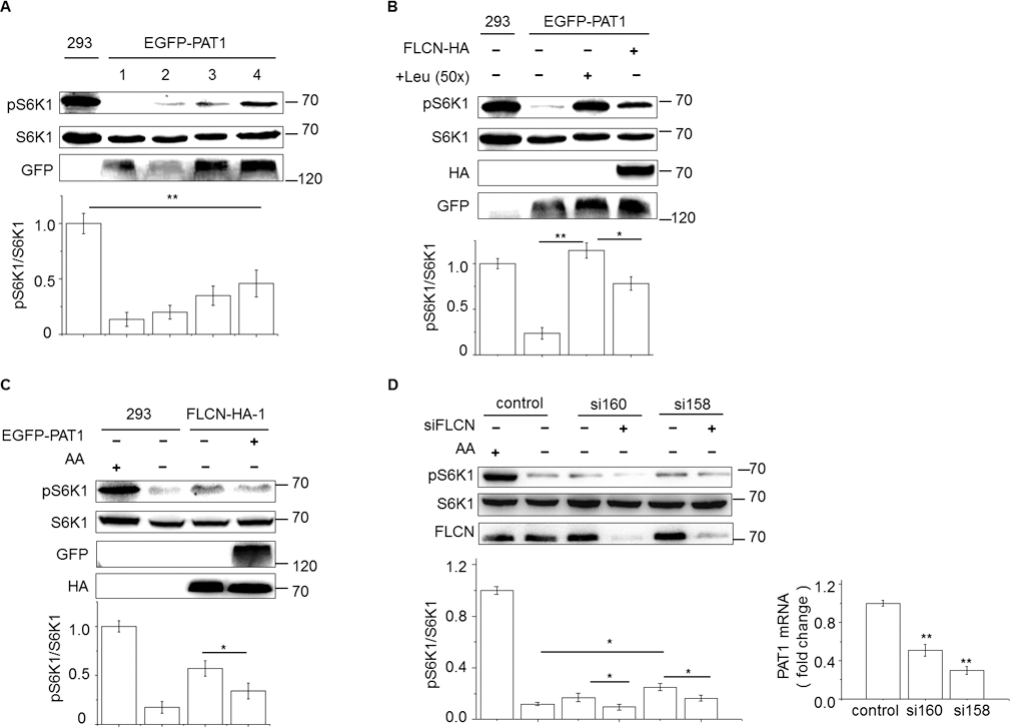
FLCN and PAT1 antagonize each other to control mTORC1. (A) Overexpression of PAT1 inhibits mTORC1. Cells were starved for 50 mins, followed by re-stimulation with complete medium for 10 mins. Three stable lines and one transient expression sample (lane 5) of EGFP-PAT1 were analysed. The predicated molecular weight of EGFP-PAT1 is about 75 kD. Due to post-translational modifications of membrane proteins, it often displays additional large shifted bands in the immunoblotting results (see Fig 5). (B-D) PAT1 counteracts FLCN for mTORC1 induction. In all experiments, transient expression (FLCN-HA or EGFP-PAT1) was lasted for 48 hrs; siRNA (FLCN or PAT1) was lasted for 36 hrs before the analysis. The siRNAs of FLCN and PAT1 have been tested before [21, 43]. Error bars denote the mean ± S.E.M. (n = 3 repeated experiments); * P<0.05, ** P<0.01, Student’s t-test.

We first confirmed that the overexpression of PAT1 inhibited mTORC1. HEK293 cells either stably or transiently expressing an EGFP-PAT1 fusion protein under the control of the CMV promoter were starved of amino acids for 50 minutes, followed by re-stimulation with amino acids for 10 minutes. Following this treatment, the level of pS6K1 was decreased in all PAT1-overexpressing cell populations (**Fig 4A**). Next, we added in the medium with high levels of leucine. We found that this treatment prevented the decrease of pS6K1 in the PAT1-overexpressing cells (**Fig 4B**; lane 4), indicating that leucine is probably the major cargo of PAT1 to stimulate mTORC1. Interestingly, a similar rescue effect was obtained by the overexpression of FLCN (**Fig 4B**; lane 5). In a reciprocal fashion, the pS6K1 remained stable upon starvation in cells expressing ectopic FLCN-HA but not in the same cells co-expressing EGFP-PAT1 (**Fig 4C**). Together, these results suggest that FLCN and PAT1 antagonize each other to regulate mTORC1. Consistent with this conclusion, the knockdown of PAT1 by two different sets of siRNA somehow inhibited the starvation-induced decrease of pS6K1 (**Fig 4D**; lane 2, 3, 5), which could be due to attenuated leucine loss; nevertheless, this effect was counteracted by the co-suppression of FLCN (**Fig 4D**; lane 4, 6).

The PAT gene family contains four member (PAT1-4). Among them, PAT1 is mostly related to PAT4 based on sequence similarities. Moreover, PAT4 has been shown to positively regulate mTORC1 [30]. In a similar experiment of Fig 4D, we found that FLCN did not counteract PAT4 in the regulation of mTORC1 (**S1 Fig**, lane 4, 6), confirming certain specificity of FLCN on the PAT genes.

### FLCN inhibits the accumulation of PAT1 on lysosome

**Fig 5:**
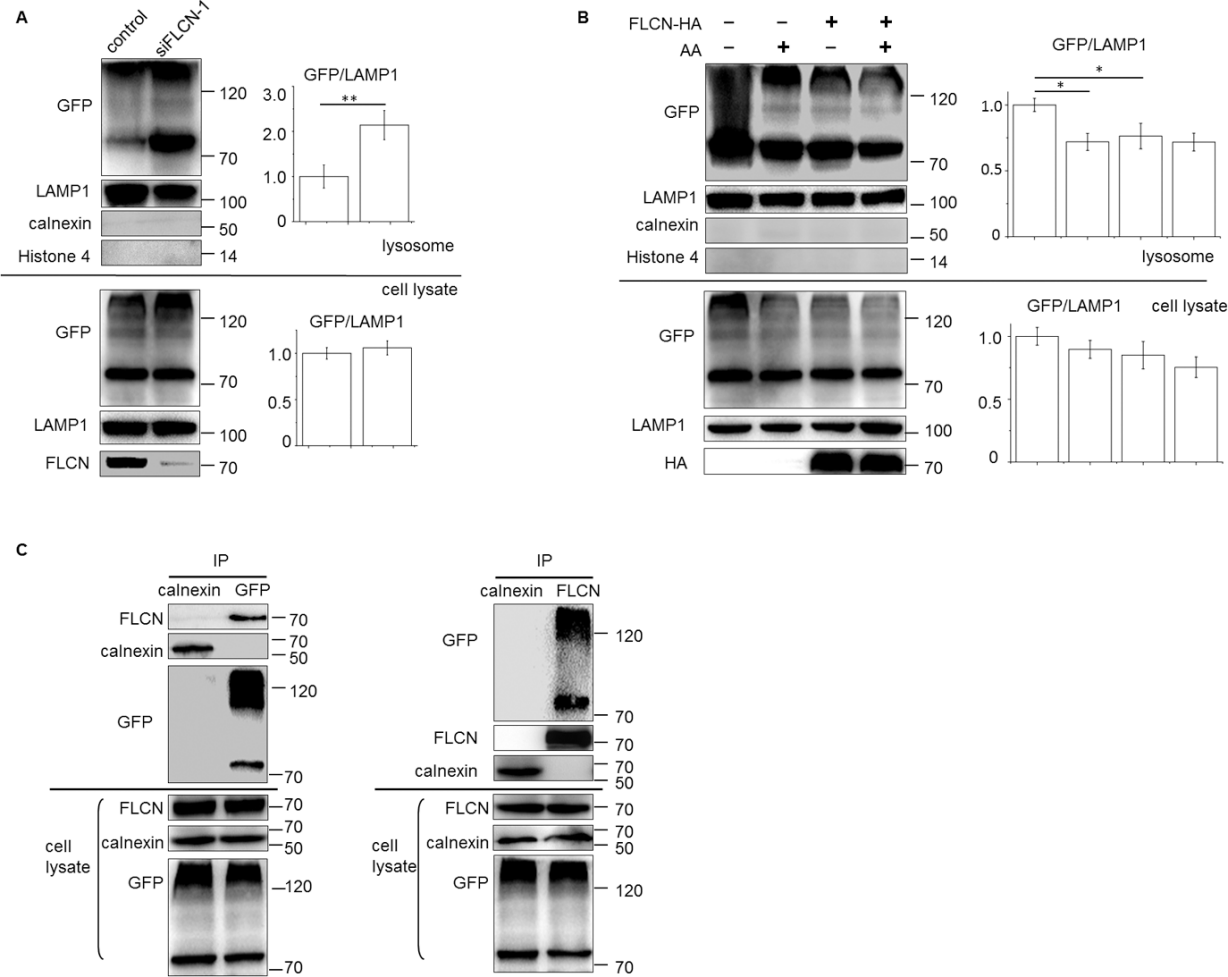
The abundance of PAT1 on lysosome is sensitive to the FLCN expression levels and nutrient status. (A, B) whole cell lysate or the purified lysosomes of the EGFP-PAT1 stable cells were immunoblotted by the indicated antibody. Starvation with amino acids was lasted for 50 mins. (C) FLCN co-immunoprecipitates with EGFP-PAT1 in a reciprocal manner. Whole cell lysate or the immunoprecipitates with the indicated antibody from the EGFP-PAT1 stable cells were analysed by western blotting. Calnexin (an ER protein) was taken as a negative control. Error bars denote the mean ± S.E.M. (n = 3 repeated experiments); * P<0.05, ** P<0.01, Student’s t-test.

FLCN may either inhibit the transport activity or abundance of PAT1, especially on the lysosomal surface. We selected one stable line expressing a relatively low level of EGFP-PAT1 to monitor PAT1 and checked its abundance on the purified lysosome by western blotting. We found that the lysosome-anchored EGFP-PAT1 in the FLCN-depleted cells was significantly increased compared with that in wild-type cells (**Fig 5A**). In contrast, the total EGFP-PAT1 prepared from the whole lysate of FLCN-depleted cells was slightly elevated. Similar results were obtained by using a different set of siFLCN (siFLCN-2 in **S2 Fig**). These results suggest that FLCN inhibits the lysosomal localization of PAT1 and may also affect the stability of PAT1 protein. Consistent with these findings, the budding yeast LST7 gene, a homologue of FLCN, has been shown to block the translocation of the general amino acid permease Gap1p to yeast lysosome-like vacuole and suppress the expression of several amino acid permeases [40, 41].

Because the intracellular localizations of several related genes, including FLCN, yeast LST7 and Gap1p, are all responsive to the nutrient status [21, 29, 40], we suspect that PAT1 may exhibit a similar dynamic pattern within the cells. In support of this hypothesis, we found that starvation induced the accumulation of EGFP-PAT1 on the lysosomal fractionation (**Fig 5B**; lane 1, 2). Thus, the binding of PAT1 to the lysosome correlates with the nutrient status in a manner similar to that of FLCN or the yeast counterpart Gap1p. Interestingly, ectopic FLCN efficiently inhibited the starvation-induced accumulation of EGFP-PAT1 on the lysosome (**Fig 5B**; lane 3, 4), implying that FLCN inhibits the starvation-induced recruitment of PAT1 on lysosome. These findings could explain the previous data of the vibrations of the lysosomal leucine levels. When the amino acid supply is limited, cells recruit PAT1 to the lysosomal surface to recycle the luminal storage, resulting in a decreased leucine level (**Fig 3D**). However, this movement of PAT1 can be inhibited by ectopic FLCN. As a result, the leucine level in the lysosome was maintained (**Fig 3D**).

As a further evidence of the close relations between FLCN and PAT1, we found that EGFP-PAT1 co-immunoprecipitated with FLCN in the cell lysate in a reciprocal manner (**Fig 5C**). Moreover, such interaction seemed not to be affected by the nutrient status (**S3 Fig**). These results suggest that FLCN may regulate the localization of PAT1 through direct interactions. Currently, we are investigating the mechanisms that govern the dynamic distribution of PAT1 by FLCN. Because FLCN has been shown to contain a distant DENN domain and thus was suspected to be a modulator of the Rab family proteins [19], we suspect that FLCN may be involved in the vesicular trafficking process of PAT1. Alternatively, FLCN may affect the transport activity of PAT1. We could not exclude this possibility yet.

## Discussion

Previously, people have shown that the depletion of FLCN failed to activate mTORC1 in the presence of amino acids (1X) and consequently regarded FLCN as a signal transducer. We herein describe a new more upstream role of FLCN in maintaining the leucine level in the lysosome. In the absence of FLCN, the concentration difference across the lysosomal membrane tends to drive leucine out of the lumen. This trend can be stopped by supplementation with high levels of leucine, and this effect seems to be facilitated by low-affinity systems and/or passive diffusion. In support of the low-affinity systems, we found that incubation with high levels of leucine requires longer time than the common amino acid stimulation treatment to activate mTORC1 in FLCN-deficient cells (up to 2 hrs *vs*. less than 30 mins, Figs 1A and 2C). In contrast, 30 mins of incubation is sufficient to activate mTORC1 in the PAT1-overexpressing cells (Fig 4B). This observation also indirectly supports that PAT1 is a transporter of leucine.

PAT1 has been found to on both the lysosome and cell surface [39, 42–44]. The lysosome-anchored PAT1 inhibits mTORC1 by attenuating the leucine signal, whereas at other sites, including the plasma membrane, it may promote mTORC1 via the bulk uptake of amino acids into the cell. Our data reveal that the intracellular localization of PAT1 is dependent on the nutrient status and FLCN expression levels. When the amino acids supply is limited, PAT1 translocates to lysosome to export luminal storage into the cytosol to meet the cell’s nutrient requirements. Conversely, decreased leucine fails to stimulate mTORC1. In this way, cells can adjust their metabolism to the nutrient conditions. The mechanism by which amino acids guide the translocation of PAT1 and FLCN remains unexplored.

Previously, one research group reported a different observation in which elevated PAT1 promoted mTORC1 activity in HEK293 cells [30, 43]. We noticed that the author stimulated the starved cells with complete medium for 30 mins. In contrast, we and others [6] performed the re-stimulation for 10 mins. With this shorter stimulation treatment, a significant amount of PAT1 may still be anchored on the lysosome, which is sufficient to maintain a low leucine signal.

A number of budding yeast genes, their mammalian homologues have been characterized to be components of mTOR signalling, are involved in a common pathway that regulates the anchorage of the general amino acid transporter Gap1p on the yeast lysosome-like vacuole. These genes include LST7 (FLCN), LST4 (FNIP1), LST8 (component of mTORC1/2), Gtr1/2 (Rag GTPases,) and SEC13 (component of the GAP complex towards Rag GTPases) [40, 45–47]. Thus, certain ancient machinery may control the nutrient signal in the lysosome by adjusting the spatial location of amino acid transporters. To elucidate this point, several questions require further investigations. For example, do these mammalian members regulate the amino acid storage in lysosome? Do they control the amino acid transporters and how about the specificities of their targets? Two recent works demonstrate that the transfer of the arginine signal to mTORC1 requires the lysosome-associated arginine transporter SLC38A9 [48, 49]. In contrast, we found that a high dose of either arginine or glutamine failed to rescue the FLCN-loss phenotype in either mammalian cells or *Drosophila*, confirming the existence of distinct mechanisms in the transduction of amino acid signals [32].

In summary, our work reveals a new role of FLCN and demonstrates that the modulation of the leucine level in the lysosome is an important mechanism to regulate mTORC1 signaling. In our provisional model, multiple factors, including the intracellular localizations, the expression levels of FLCN and PAT1, and the local nutrient conditions, can all affect the leucine signal in the lysosome and thereby the mTORC1 activity. This work extends our knowledge of how cells sense the available nutrients and also helps us to appreciate the context-dependent relationships between FLCN and mTORC1.

## Supporting Information

S1 Fig FLCN does not counteract PAT4 in the regulation of mTORC1. The experiment was performed similarly as that in Fig 4D. The two different sets of siPAT4, including si435 and si437, have been described before [30]. The knockdown efficiencies of both siPAT1s were analyzed by qRT-PCR and shown to the right.
(TIF)

S2 Fig Suppression of FLCN (using a second siFLCN) promotes the accumulation of PAT1 on the lysosome. siFLCN-2 is different with the one used in Fig 5A. Both siFLCN-1 and siFLCN-2 have been described before [21].
(TIF)

S3 Fig Nutrient status has little effects to influence the interactions between the endogenous FLCN and EGFP-PAT1. Left: Cell lysates of EGFP-PAT1 stable cells were co-immunoprecipitated with the antibody against either calnexin (negative control) or FLCN; Right: the opposite direction of the co-IP experiment, the 293 cells transfected with an empty EGFP plasmid was taken as a negative control. Note with or without amino acids, the strength of interactions (marked with stars) did not show significant differences.
(TIF)

## References

[1] Laplante M, Sabatini DM (2012) mTOR Signaling in Growth Control and Disease. Cell 149: 274–293. 10.1016/j.cell.2012.03.017 22500797 PMC3331679

[2] Albert V, Hall MN (2015) mTOR signaling in cellular and organismal energetics. Current opinion in cell biology 33: 55–66. 10.1016/j.ceb.2014.12.001 25554914

[3] Sancak Y, Peterson TR, Shaul YD, Lindquist RA, Thoreen CC, Bar-Peled L, et al (2008) The Rag GTPases bind raptor and mediate amino acid signaling to mTORC1. Science 320: 1496–1501. 10.1126/science.1157535 18497260 PMC2475333

[4] Kim E, Goraksha-Hicks P, Li L, Neufeld TP, Guan KL (2008) Regulation of TORC1 by Rag GTPases in nutrient response. Nat Cell Biol 10: 935–945. 10.1038/ncb1753 18604198 PMC2711503

[5] Sancak Y, Bar-Peled L, Zoncu R, Markhard AL, Nada S, Sabatini DM (2010) Ragulator-Rag complex targets mTORC1 to the lysosomal surface and is necessary for its activation by amino acids. Cell 141: 290–303. 10.1016/j.cell.2010.02.024 20381137 PMC3024592

[6] Zoncu R, Bar-Peled L, Efeyan A, Wang S, Sancak Y, Sabatini DM (2011) mTORC1 senses lysosomal amino acids through an inside-out mechanism that requires the vacuolar H(+)-ATPase. Science 334: 678–683. 10.1126/science.1207056 22053050 PMC3211112

[7] Bar-Peled L, Schweitzer LD, Zoncu R, Sabatini DM (2012) Ragulator is a GEF for the rag GTPases that signal amino acid levels to mTORC1. Cell 150: 1196–1208. 10.1016/j.cell.2012.07.032 22980980 PMC3517996

[8] Betz C, Hall MN (2013) Where is mTOR and what is it doing there? The Journal of cell biology 203: 563–574. 10.1083/jcb.201306041 24385483 PMC3840941

[9] Menon S, Dibble CC, Talbott G, Hoxhaj G, Valvezan AJ, Takahashi H, et al (2014) Spatial control of the TSC complex integrates insulin and nutrient regulation of mTORC1 at the lysosome. Cell 156: 771–785. 10.1016/j.cell.2013.11.049 24529379 PMC4030681

[10] Schmidt LS, Warren MB, Nickerson ML, Weirich G, Matrosova V, Toro JR, et al (2001) Birt-Hogg-Dube syndrome, a genodermatosis associated with spontaneous pneumothorax and kidney neoplasia, maps to chromosome 17p11.2. American journal of human genetics 69: 876–882. 11533913 10.1086/323744 PMC1226073

[11] Nickerson ML, Warren MB, Toro JR, Matrosova V, Glenn G, Turner ML, et al (2002) Mutations in a novel gene lead to kidney tumors, lung wall defects, and benign tumors of the hair follicle in patients with the Birt-Hogg-Dube syndrome. Cancer Cell 2: 157–164. 12204536 10.1016/s1535-6108(02)00104-6

[12] Schmidt LS (2013) Birt-Hogg-Dube syndrome: from gene discovery to molecularly targeted therapies. Familial cancer 12: 357–364. 10.1007/s10689-012-9574-y 23108783 PMC3637987

[13] Tee AR, Pause A (2013) Birt-Hogg-Dube: tumour suppressor function and signalling dynamics central to folliculin. Familial cancer 12: 367–372. 10.1007/s10689-012-9576-9 23096221

[14] Singh SR, Zhen W, Zheng Z, Wang H, Oh SW, Liu W, et al (2006) The *Drosophila* homolog of the human tumor suppressor gene BHD interacts with the JAK-STAT and Dpp signaling pathways in regulating male germline stem cell maintenance. Oncogene 25: 5933–5941. 16636660 10.1038/sj.onc.1209593

[15] Hong SB, Oh H, Valera VA, Stull J, Ngo DT, Baba M, et al (2010) Tumor suppressor FLCN inhibits tumorigenesis of a FLCN-null renal cancer cell line and regulates expression of key molecules in TGF-beta signaling. Molecular cancer 9: 160 10.1186/1476-4598-9-160 20573232 PMC2907329

[16] Cash TP, Gruber JJ, Hartman TR, Henske EP, Simon MC (2011) Loss of the Birt-Hogg-Dube tumor suppressor results in apoptotic resistance due to aberrant TGFbeta-mediated transcription. Oncogene 30: 2534–2546. 10.1038/onc.2010.628 21258407 PMC3109270

[17] Goncharova EA, Goncharov DA, James ML, Atochina-Vasserman EN, Stepanova V, Hong SB, et al (2014) Folliculin controls lung alveolar enlargement and epithelial cell survival through E-cadherin, LKB1, and AMPK. Cell reports 7: 412–423. 10.1016/j.celrep.2014.03.025 24726356 PMC4034569

[18] Khabibullin D, Medvetz DA, Pinilla M, Hariharan V, Li C, Hergrueter A, et al (2014) Folliculin regulates cell-cell adhesion, AMPK, and mTORC1 in a cell-type-specific manner in lung-derived cells. Physiol Rep 2(8) pii: e12107 10.14814/phy2.12107 25121506 PMC4246594

[19] Nookala RK, Langemeyer L, Pacitto A, Ochoa-Montano B, Donaldson JC, Blaszczyk BK, et al (2012) Crystal structure of folliculin reveals a hidDENN function in genetically inherited renal cancer. Open biology 2: 120071 10.1098/rsob.120071 22977732 PMC3438538

[20] Dunlop EA, Seifan S, Claessens T, Behrends C, Kamps MA, Rozycka E, et al (2014) FLCN, a novel autophagy component, interacts with GABARAP and is regulated by ULK1 phosphorylation. Autophagy 10: 1749–1760. 10.4161/auto.29640 25126726 PMC4198360

[21] Petit CS, Roczniak-Ferguson A, Ferguson SM (2013) Recruitment of folliculin to lysosomes supports the amino acid-dependent activation of Rag GTPases. The Journal of cell biology 202: 1107–1122. 10.1083/jcb.201307084 24081491 PMC3787382

[22] Hasumi H, Baba M, Hasumi Y, Huang Y, Oh H, Hughes RM, et al (2012) Regulation of mitochondrial oxidative metabolism by tumor suppressor FLCN. J Natl Cancer Inst 104: 1750–1764. 10.1093/jnci/djs418 23150719 PMC3502196

[23] Okimoto K, Sakurai J, Kobayashi T, Mitani H, Hirayama Y, Nickerson ML, et al (2004) A germ-line insertion in the Birt-Hogg-Dube (BHD) gene gives rise to the Nihon rat model of inherited renal cancer. Proc Natl Acad Sci USA 101: 2023–2027. 14769940 10.1073/pnas.0308071100 PMC357045

[24] Baba M, Hong SB, Sharma N, Warren MB, Nickerson ML, Iwamatsu A, et al (2006) Folliculin encoded by the BHD gene interacts with a binding protein, FNIP1, and AMPK, and is involved in AMPK and mTOR signaling. Proc Natl Acad Sci USA 103:15552–15557. 17028174 10.1073/pnas.0603781103 PMC1592464

[25] Baba M, Furihata M, Hong SB, Tessarollo L, Haines DC, Southon E, et al (2008) Kidney-targeted Birt-Hogg-Dubé gene inactivation in a mouse model: Erk1/2 and Akt-mTOR activation, cell hyperproliferation, and polycystic kidneys. J Natl Cancer Inst 100:140–154. 10.1093/jnci/djm288 18182616 PMC2704336

[26] Luijten MN, Basten SG, Claessens T, Vernooij M, Scott CL, Janssen R, et al (2013) Birt-Hogg-Dube syndrome is a novel ciliopathy. Hum Mol Genet 22: 4383–4397. 10.1093/hmg/ddt288 23784378 PMC3792695

[27] Possik E, Jalali Z, Nouet Y, Yan M, Gingras MC, Schmeisser K, et al (2014) Folliculin regulates ampk-dependent autophagy and metabolic stress survival. PLoS Genet 10: e1004273 10.1371/journal.pgen.1004273 24763318 PMC3998892

[28] Liu W, Chen Z, Ma Y, Wu X, Jin Y, Liu W (2013) Genetic characterization of the *Drosophila* birt-hogg-dube syndrome gene. PloS one 8: e65869 10.1371/journal.pone.0065869 23799055 PMC3684598

[29] Tsun ZY, Bar-Peled L, Chantranupong L, Zoncu R, Wang T, Kim C, et al (2013) The folliculin tumor suppressor is a GAP for the RagC/D GTPases that signal amino acid levels to mTORC1. Mol Cell 52: 495–505. 10.1016/j.molcel.2013.09.016 24095279 PMC3867817

[30] Heublein S, Kazi S, Ogmundsdottir MH, Attwood EV, Kala S, Boyd CA, et al Proton-assisted amino-acid transporters are conserved regulators of proliferation and amino-acid-dependent mTORC1 activation. Oncogene 29, 4068–4079 (2010). 10.1038/onc.2010.177 20498635 PMC3018277

[31] Liu B, Du H, Rutkowski R, Gartner A, Wang X (2012) LAAT-1 is the lysosomal lysine/arginine transporter that maintains amino acid homeostasis. Science 337: 351–354. 10.1126/science.1220281 22822152 PMC3432903

[32] Jewell JL, Kim YC, Russell RC, Yu FX, Park HW, Plouffe SW, et al (2015) Differential regulation of mTORC1 by leucine and glutamine. Science 347: 194–198. 10.1126/science.1259472 25567907 PMC4384888

[33] Bowman EJ, Siebers A, Altendorf K (1988) Bafilomycins: a class of inhibitors of membrane ATPases from microorganisms, animal cells, and plant cells. Proc Natl Acad Sci USA 85: 7972–7976. 2973058 10.1073/pnas.85.21.7972 PMC282335

[34] Jewell JL, Russell RC, Guan KL (2013) Amino acid signalling upstream of mTOR. Nat Rev Mol Cell Biol 14: 133–139. 10.1038/nrm3522 23361334 PMC3988467

[35] Berra E, Forcella M, Giacchini R, Parenti P (2006) Leucine transport across plasmamembranes from the scud Echinogammarus stammeri (Amphipoda: Gammaridae). Ann Limnol—Int J Lim 42: 79–85.

[36] Fiandra L, Caccia S, Giordana B, Casartelli M (2010) Leucine transport by the larval midgut of the parasitoid Aphidius ervi (Hymenoptera). J Insect Physiol 56: 165–169. 10.1016/j.jinsphys.2009.09.015 19799906

[37] Thwaites DT, Anderson CM (2011) The SLC36 family of proton-coupled amino acid transporters and their potential role in drug transport. Br J Pharmacol 164: 1802–1816. 10.1111/j.1476-5381.2011.01438.x 21501141 PMC3246705

[38] Neufeld TP (2004) Genetic analysis of TOR signaling in *Drosophila*. Curr Top Microbiol Immunol 279: 139–152. 14560956 10.1007/978-3-642-18930-2_9

[39] Sagne C, Agulhon C, Ravassard P, Darmon M, Hamon M, El Mestikawy S, et al (2001) Identification and characterization of a lysosomal transporter for small neutral amino acids. Proc Natl Acad Sci USA 98: 7206–7211. 11390972 10.1073/pnas.121183498 PMC34647

[40] Roberg KJ, Bickel S, Rowley N, Kaiser CA (1997) Control of amino acid permease sorting in the late secretory pathway of Saccharomyces cerevisiae by SEC13, LST4, LST7 and LST8. Genetics 147: 1569–1584. 9409822 10.1093/genetics/147.4.1569 PMC1208332

[41] van Slegtenhorst M, Khabibullin D, Hartman TR, Nicolas E, Kruger WD, Henske EP (2007) The Birt-Hogg-Dube and tuberous sclerosis complex homologs have opposing roles in amino acid homeostasis in Schizosaccharomyces pombe. J Biol Chem 282: 24583–24590. 17556368 10.1074/jbc.M700857200

[42] Wreden CC, Johnson J, Tran C, Seal RP, Copenhagen DR, Reimer RJ, et al (2003) The H+-coupled electrogenic lysosomal amino acid transporter LYAAT1 localizes to the axon and plasma membrane of hippocampal neurons. J Neurosci 23: 1265–1275. 12598615 10.1523/JNEUROSCI.23-04-01265.2003 PMC6742289

[43] Ogmundsdottir MH, Heublein S, Kazi S, Reynolds B, Visvalingam SM, Shaw MK, et al (2012) Proton-assisted amino acid transporter PAT1 complexes with Rag GTPases and activates TORC1 on late endosomal and lysosomal membranes. PLoS One 7, e36616 10.1371/journal.pone.0036616 22574197 PMC3344915

[44] Chen Z, Fei YJ, Anderson CM, Wake KA, Miyauchi S, Huang W, et al (2003) Structure, function and immunolocalization of a proton-coupled amino acid transporter (hPAT1) in the human intestinal cell line Caco-2. J Physiol 546: 349–361. 12527723 10.1113/jphysiol.2002.026500 PMC2342508

[45] Gao M, Kaiser CA (2006) A conserved GTPase-containing complex is required for intracellular sorting of the general amino-acid permease in yeast. Nat Cell Biol 8: 657–667. 16732272 10.1038/ncb1419

[46] Bar-Peled L, Chantranupong L, Cherniack AD, Chen WW, Ottina KA, Grabiner BC, et al (2013) A Tumor suppressor complex with GAP activity for the Rag GTPases that signal amino acid sufficiency to mTORC1. Science 340: 1100–1106. 10.1126/science.1232044 23723238 PMC3728654

[47] Levine TP, Daniels RD, Gatta AT, Wong LH, Hayes MJ (2013) The product of C9orf72, a gene strongly implicated in neurodegeneration, is structurally related to DENN Rab-GEFs. Bioinformatics 29: 499–503. 10.1093/bioinformatics/bts725 23329412 PMC3570213

[48] Rebsamen M, Pochini L, Stasyk T, de Araujo ME, Galluccio M, Kandasamy RK, et al (2015) SLC38A9 is a component of the lysosomal amino acid sensing machinery that controls mTORC1. Nature 519: 477–481. 10.1038/nature14107 25561175 PMC4376665

[49] Wang S, Tsun ZY, Wolfson RL, Shen K, Wyant GA, Plovanich ME, et al (2015) Lysosomal amino acid transporter SLC38A9 signals arginine sufficiency to mTORC1. Science 347, 188–194. 10.1126/science.1257132 25567906 PMC4295826

