# Supplementary figures and images for: FLCN Maintains the Leucine Level in Lysosome to Stimulate mTORC1
Source: PLoS One. 2016 Jun 9;11(6):e0157100. doi: 10.1371/journal.pone.0157100 (PMC4900541; doi:10.1371/journal.pone.0157100)

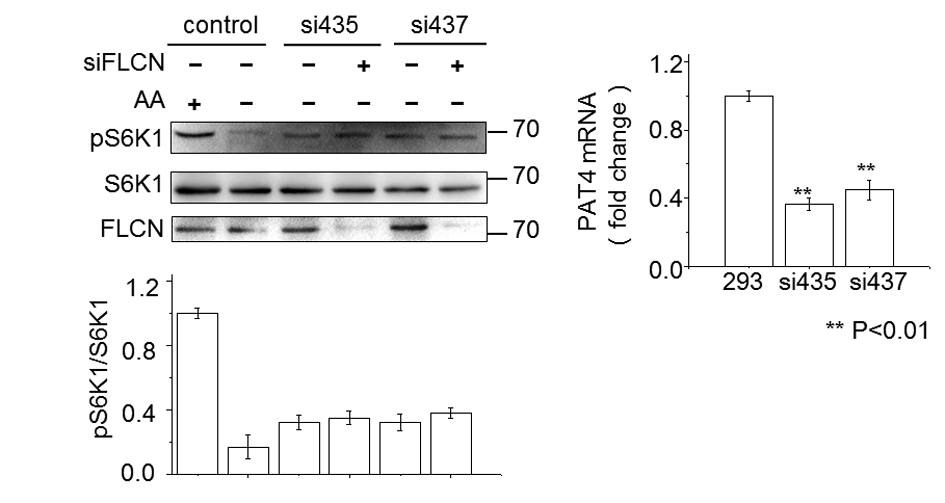

Supplement: S1 Fig — The experiment was performed similarly as that in Fig 4D. The two different sets of siPAT4, including si435 and si437, have been described before [30]. The knockdown efficiencies of both siPAT1s were analyzed by qRT-PCR and shown to the right. (TIF) [file pone.0157100.s001.tif]

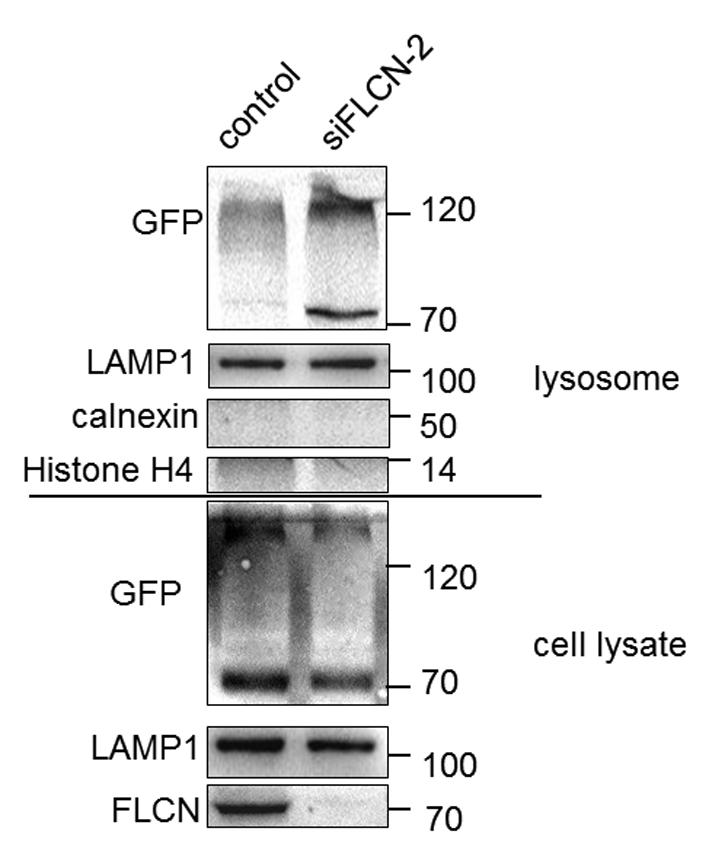

Supplement: S2 Fig — siFLCN-2 is different with the one used in Fig 5A. Both siFLCN-1 and siFLCN-2 have been described before [21]. (TIF) [file pone.0157100.s002.tif]

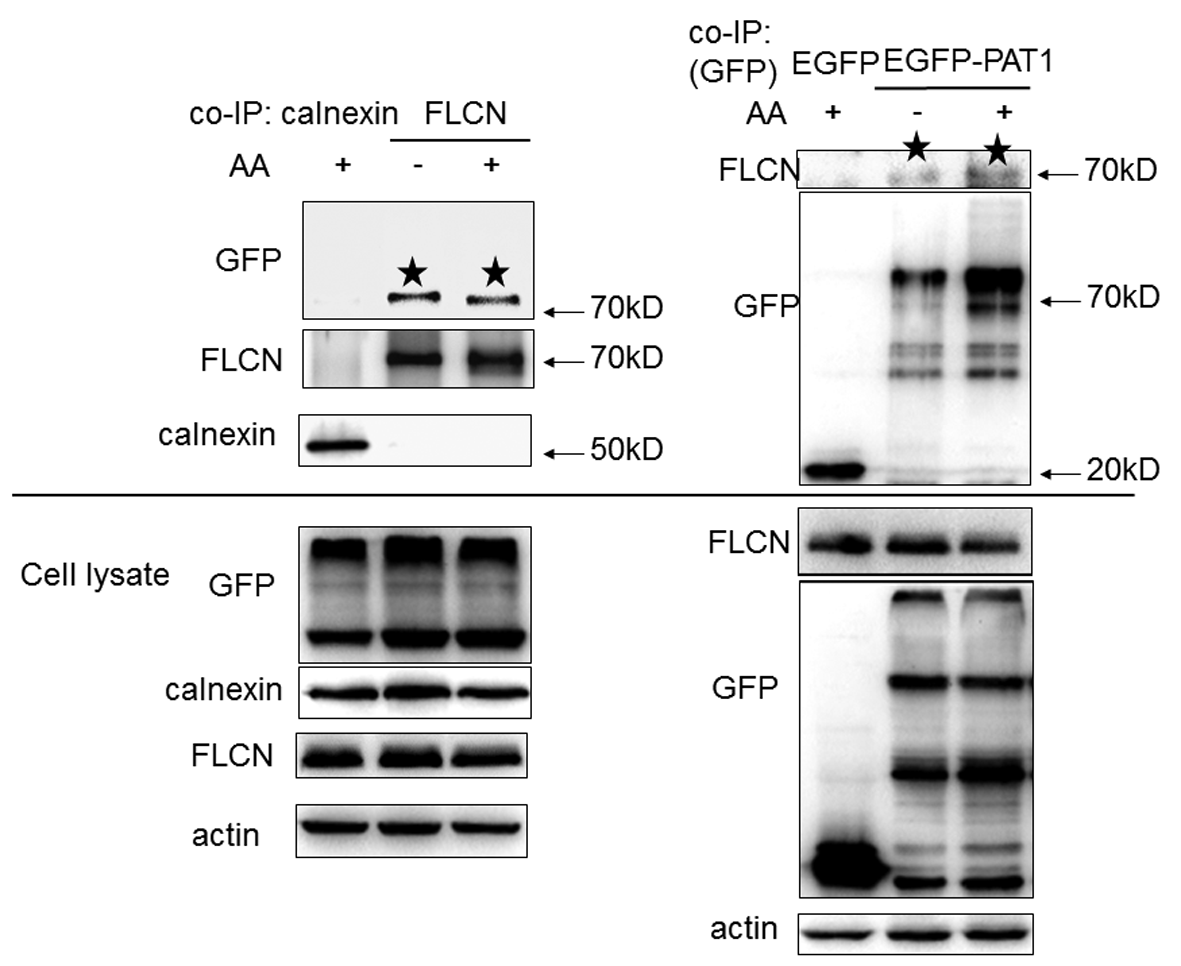

Supplement: S3 Fig — Left: Cell lysates of EGFP-PAT1 stable cells were co-immunoprecipitated with the antibody against either calnexin (negative control) or FLCN; Right: the opposite direction of the co-IP experiment, the 293 cells transfected with an empty EGFP plasmid was taken as a negative control. Note with or without amino acids, the strength of interactions (marked with stars) did not show significant differences. (TIF) [file pone.0157100.s003.tif]
